# Neighborhood Deprivation and Voice and Reflux Symptom Burden in a Tertiary Laryngology Cohort

**DOI:** 10.1002/lary.70610

**Published:** 2026-05-06

**Authors:** Sandra Stinnett, Shirley X. Liu, Katie M. Carlson, Leah Helou, Libby Smith, Chloe Santa Maria, Angela L. Mazul

**Affiliations:** ^1^ University of Pittsburgh School of Medicine Pittsburgh Pennsylvania USA; ^2^ Department of Otolaryngology‐Head and Neck Surgery University of Pittsburgh School of Medicine Pittsburgh Pennsylvania USA; ^3^ Cancer Epidemiology and Prevention Program UPMC Hillman Cancer Center Pittsburgh Pennsylvania USA; ^4^ University of Pittsburgh School of Communication Science Disorders Pittsburgh Pennsylvania USA

**Keywords:** allostatic load, area deprivation index, functional laryngeal disorders, patient‐reported outcome measures

## Abstract

**Objective:**

The voice handicap index‐10 (VHI‐10) and reflux symptoms index (RSI) are validated, patient‐reported outcome measures (PROMs) commonly used in laryngology to assess the subjective impact of voice disorders and severity of symptoms associated with laryngopharyngeal reflux, respectively. This study aims to evaluate the relationship between neighborhood‐level socioeconomic disadvantage, as measured by the area deprivation index (ADI), and patient‐reported laryngeal outcomes.

**Methods:**

An analysis of 1310 adult patients who were referred to a tertiary care laryngology clinic between January and December 2024 was conducted. Patient addresses were geocoded and matched to corresponding ADI national percentile scores, and internally derived ADI quartiles were formed. Median VHI‐10 and RSI scores by ADI quartile were compared using the Kruskal‐Wallis test. Multivariable linear regression was performed to investigate the association between ADI and VHI‐10 or RSI scores, adjusting for sociodemographic (race, sex, and age) and clinical (comorbidities, depression/anxiety, insurance status, BMI, and primary diagnosis) factors.

**Results:**

Patients living in the least deprived ADI quartile had significantly lower median VHI‐10 and RSI Scores, compared to patients in the most deprived ADI quartile (10 vs. 14, and 15 vs. 20, respectively). In adjusted analysis, living in a higher ADI area was significantly associated with a higher VHI‐10 (*β* = 0.04, 95% confidence interval [CI]: 0.008–0.06) or RSI (*β* = 0.04, 95% CI: 0.01–0.07) score.

**Conclusions:**

Higher neighborhood socioeconomic deprivation is significantly associated with higher VHI‐10 and RSI scores. Patients living in more deprived areas may face barriers to accessing care, including low health literacy and limited resources.

**Level of Evidence:**

3.

## 1 Introduction

Laryngeal symptoms are highly prevalent in the general population and represent a substantial public health burden. In 2012, population‐based studies estimated that voice disorders affect approximately 6%–9% of adults at any given time and up to 30% of individuals across the lifespan [1, 2, 3]. In 2022, the number of annually reported voice problems among Americans increased to 12.2% of the population, with higher prevalence among women, older adults, professional voice users and individuals with medical and psychological comorbidities [4]. Because many laryngeal disorders lack a single objective biomarker and symptom severity often correlates poorly with laryngoscopic findings, patient reported outcome measures (PROMs) have been central to laryngologic evaluation. Instruments such as the voice handicap index‐10 (VHI‐10) and the reflux symptoms index (RSI) provide standardized measurements of perceived voice handicap and reflux symptoms, respectively, and are widely used for screening, severity stratification, treatment selection and longitudinal monitoring of therapeutic response [5, 6, 7]. The interplay of socioeconomic and environmental stressors may amplify both exposure risk and physiologic vulnerability, thus contributing to disparities in prevalence, severity and subjective impact of laryngeal symptoms across patient populations [8].

Social determinants of health (SDoH), defined as the conditions in which people are born, grow, live, work and age, play a central role in shaping health outcomes, healthcare access and disease burden across populations [9]. The World Health Organization (WHO) has long recognized SDoH as a primary driver of health inequities worldwide, influencing morbidity, mortality and quality of life through structural, economic and environmental mechanisms [9]. In the United States, socioeconomic disadvantage has been consistently linked to delayed access to care, increased disease severity at presentation and poorer patient‐reported outcomes across multiple medical and surgical subspecialties [8, 10, 11]. Within otolaryngology, healthcare disparities have been described in head and neck cancer outcomes, pediatric airway disease, hearing loss, and sleep‐disordered breathing [12, 13, 14]. Despite the centrality of PROMs in clinical laryngology, the influence of neighborhood‐level socioeconomic context on patient‐reported laryngeal symptom burden remains under‐characterized.

Multi‐level equity sciences is a novel approach to addressing health disparities, considering both individual‐level and exposome impacts on wellbeing [15]. These direct and cumulative biologic effects on human physiology through chronic activation of stress response pathways are a phenomenon termed allostatic load [16, 17]. Though SDoH is widely recognized as a driver of health inequities on an individual level, neighborhood‐level socioeconomic deprivation is a powerful underlying factor in the pathophysiologic impact of these stressors leading to increased allostatic load. Area deprivation index (ADI) is a validated, census‐based composite measure that quantifies neighborhood disadvantage using income, education, employment and housing quality metrics. Higher ADI is associated with elevated biologic stress markers of allostatic load, making it not only a proxy for access to care, but also a surrogate for cumulative biologic stress exposure that may directly shape expression and symptom perception [18, 19, 20].

PROMs such as VHI‐10 and RSI remain foundational to the diagnostic evaluation, risk stratification, and longitudinal management of voice and reflux‐related disorders [5, 6, 7]. However, neighborhood‐level deprivation and its association with PROM scores remain largely unexplored. Patients living in more deprived neighborhoods may have both external structural barriers to care and greater internal biologic vulnerability, together potentially contributing to amplified symptom severity upon presentation. The objective of this study is to examine the association between neighborhood‐level socioeconomic deprivation, as measured by ADI, and patient‐reported laryngeal outcomes using VHI‐10 and RSI scores in a large tertiary‐care laryngology cohort. We hypothesize that higher ADI will be independently associated with worse VHI‐10 and RSI scores, reflecting the combined influence of healthcare access disparities and potential stress‐mediated physiologic vulnerability.

## 2 Materials and Methods

### 2.1 Study Design and Population

This was a single‐center cross‐sectional study of adult patients (≥ 18 years) evaluated at a tertiary‐care laryngology clinic between January 1, 2024, and December 31, 2024. Consecutive patients who completed both the VHI‐10 and the RSI at the time of initial consultation were eligible for inclusion. Clinical and sociodemographic data were collected (Table 1). Patients with incomplete PROMs, missing address data, or missing ADI were excluded. The CONSORT diagram for our study population is included in Supporting Information S1. This study was approved by the institutional review board with a waiver of informed consent due to its retrospective nature.

**TABLE 1:** Clinical and sociodemographic characteristics for the overall cohort (*n* = 1310) stratified by area deprivation index quartile.

		ADI Quartile 1‐least deprived (national rankings: 1–44)	ADI Quartile 2 (national rankings: 45–65)	ADI Quartile 3 (national rankings: 66–81)	ADI Quartile 4–most deprived (national rankings: 82–100)	*p*
		331 (25.3)	348 (26.6)	317 (24.2)	314 (24.0)	
Race (%)						< 0.001
	Black	12 (3.6)	17 (4.9)	30 (9.5)	76 (24.2)	
	Other or unknown	37 (11.2)	20 (5.7)	13 (4.1)	16 (5.1)	
	White	282 (85.2)	311 (89.4)	274 (86.4)	222 (70.7)	
Sex (%)						0.48
	Female	212 (64.0)	220 (63.2)	217 (68.5)	210 (66.9)	
	Male	118 (35.6)	128 (36.8)	100 (31.5)	104 (33.1)	
	Unknown	1 (0.3)	0 (0.0)	0 (0.0)	0 (0.0)	
Age (median [IQR])		55.5 [37.1, 69.9]	56.1 [39.4, 70.7]	59.2 [41.2, 70.1]	61.0 [44.5, 69.0]	0.32
Ever smoking status (%)						< 0.001
	No	220 (66.5)	216 (62.1)	188 (59.3)	154 (49.0)	
	Yes	107 (32.3)	129 (37.1)	127 (40.1)	160 (51.0)	
	Unknown	4 (1.2)	3 (0.9)	2 (0.6)	0 (0.0)	
BMI (median [IQR])		26.3 [22.9, 30.2]	27.6 [24.1, 32.2]	28.5 [24.7, 33.7]	29.4 [25.3, 34.8]	< 0.001
Hypertension (%)^a^						0.002
	No	250 (75.5)	243 (69.8)	217 (68.5)	197 (62.7)	
	Yes	76 (23.0)	103 (29.6)	96 (30.3)	116 (36.9)	
	Unknown	5 (1.5)	2 (0.6)	4 (1.3)	1 (0.3)	
Diabetes (%)^a^						0.004
	No	304 (91.8)	311 (89.4)	283 (89.3)	262 (83.4)	
	Prediabetes	1 (0.3)	1 (0.3)	2 (0.6)	1 (0.3)	
	Yes	22 (6.6)	34 (9.8)	28 (8.8)	50 (15.9)	
	Unknown	4 (1.2)	2 (0.6)	4 (1.3)	1 (0.3)	
Coronary artery disease (%)^a^						0.01
	No	318 (96.1)	328 (94.3)	307 (96.8)	293 (93.3)	
	Yes	9 (2.7)	18 (5.2)	6 (1.9)	20 (6.4)	
	Unknown	4 (1.2)	2 (0.6)	4 (1.3)	1 (0.3)	
Depression or anxiety (%)^a^						0.06
	No	261 (78.9)	246 (70.7)	237 (74.8)	229 (72.9)	
	Yes	66 (19.9)	100 (28.7)	76 (24.0)	84 (26.8)	
	Unknown	4 (1.2)	2 (0.6)	4 (1.3)	1 (0.3)	
Insurance status (%)						< 0.001
	Medicaid	13 (3.9)	32 (9.2)	51 (16.1)	69 (22.0)	
	Medicare	120 (36.3)	135 (38.8)	121 (38.2)	131 (41.7)	
	Other	0 (0.0)	5 (1.4)	0 (0.0)	6 (1.9)	
	Private or managed care	195 (58.9)	164 (47.1)	143 (45.1)	104 (33.1)	
	Unknown	3 (0.9)	12 (3.4)	2 (0.6)	4 (1.3)	
Primary diagnosis (%)^a^						0.02
	Benign Lesions	78 (23.6)	89 (25.6)	86 (27.1)	92 (29.3)	
	Bilateral vocal fold paralysis	1 (0.3)	1 (0.3)	2 (0.6)	0 (0.0)	
	Cancer	2 (0.6)	4 (1.1)	4 (1.3)	1 (0.3)	
	Dyspnea, cough, or throat irritation	51 (15.4)	37 (10.6)	29 (9.1)	26 (8.3)	
	Functional	128 (38.7)	115 (33.0)	100 (31.5)	101 (32.2)	
	Infectious or inflammatory	7 (2.1)	2 (0.6)	3 (0.9)	3 (1.0)	
	Neurological	28 (8.5)	46 (13.2)	56 (17.7)	50 (15.9)	
	Oropharyngeal/esophageal dysphagia	12 (3.6)	19 (5.5)	10 (3.2)	17 (5.4)	
	Not laryngeal	24 (7.3)	35 (10.1)	27 (8.5)	24 (7.6)	
Prior diagnosis of LPRD/GERD (%)						0.31
	No	278 (84.0)	304 (87.4)	276 (87.1)	279 (88.9)	
	Yes	53 (16.0)	44 (12.6)	41 (12.9)	35 (11.1)	
RSI (median [IQR])		15.0 [8.0, 22.0]	15.5 [8.0, 23.0]	17.0 [9.0, 25.0]	20.0 [11.0, 29.0]	< 0.001
VHI‐10 (median [IQR])		10.0 [2.0, 18.0]	13.0 [5.0, 23.0]	14.0 [3.0, 23.0]	14.0 [4.0, 25.0]	< 0.001

Abbreviations: ADI, area deprivation index; BMI, body mass index; GERD, gastroesophageal reflux disease; LPRD, laryngopharyngeal reflux disease; RSI, reflux severity index; VHI‐10, voice handicap index‐10.

^a^ Fisher's exact test was used due to small sample sizes.

### 2.2 Area Deprivation Index

Patient residential addresses were geocoded to their corresponding census block groups, which were then linked with the 2020 Neighborhood Atlas ADI. Developed by the University of Wisconsin, the ADI is a validated measure of neighborhood socioeconomic deprivation [12, 19]. ADI national ranking scores range from 1 to 100, with higher values representing higher levels of neighborhood deprivation. ADI quartiles were established based on the distribution of values within our study population, with ranges of 1–44 for the first quartile (least deprived), 45–65 for the second quartile, 66–81 for the third quartile, and 82–100 for the fourth quartile (most deprived).

### 2.3 Categorization of Diagnoses

Diagnoses were abstracted from the electronic medical record, including up to three encounter diagnoses per visit. Diagnoses were initially classified as laryngeal, laryngeal‐adjacent, and non‐laryngeal categories (Table 2; Supporting Information S2). Laryngeal diagnoses were then further subdivided hierarchically into meaningful subgroups to better evaluate the relationship between ADI and PROMs across diagnostic phenotypes. When the primary diagnosis reflected a laryngeal disorder, this diagnosis was used for classification. When the primary diagnosis was non‐laryngeal, the secondary or tertiary diagnosis was used if either represented a laryngeal condition. Encounters in which dysphonia or hoarseness was listed as the primary diagnosis without an associated structural or neurologic laryngeal disorder were categorized as functional laryngeal disorders. Laryngopharyngeal reflux disease (LPRD) and gastroesophageal reflux disease (GERD) were classified as a separate diagnostic category regardless of their position among listed diagnoses. Conditions were then grouped into six a priori clinical categories: functional laryngeal disorders, neurologic voice disorders, benign/structural lesions, dysphagia/upper aerodigestive dysfunction, dyspnea/cough/throat irritation, and reflux‐related disorders. Bilateral vocal fold paralysis, malignant or premalignant lesions, and infectious/inflammatory disorders were classified as “other” due to small sample size.

**TABLE 2:** Grouping of encounter primary diagnoses into laryngeal diagnostic categories.

Diagnostic categories	Conditions
Functional laryngeal disorders	Dysphonia; Muscle tension dysphonia (MTD); Hoarseness; Paradoxical vocal fold motion disorder (PVFMD); Exercise‐induced laryngeal obstruction (EILO); Inducible laryngeal obstruction (ILO); Globus sensation; Globus pharyngeus; Irritable larynx; Mutational falsetto; Gender dysphoria.
Neurologic laryngeal disorders	Unilateral vocal fold paresis; Unilateral vocal fold paralysis; Bilateral vocal fold paresis; Bilateral vocal fold paralysis; Bilateral complete vocal fold paralysis; Laryngeal spasm; Adductor spasmodic dysphonia; Laryngeal dystonia; Vocal tremor; Essential tremor; Unspecified tremor.
Benign/structural lesion	Vocal fold polyp; Vocal fold cyst; Vocal fold lesion; Vocal process granuloma; Vocal process lesion; Reinke's edema/Polypoid corditis; Vocal fold edema; Vocal fold scar; Vocal fold atrophy; Vocal fold leukoplakia; Laryngeal papilloma; Laryngeal cyst; Laryngeal mass; Larynx mass; Laryngeal edema; Lesion of pharynx; Trauma of larynx, sequela; Closed fracture of larynx and trachea; Lymphedema due to radiation; Parkinson's disease.
Dysphagia	Dysphagia (unspecified); Esophageal dysphagia; Cricopharyngeus muscle dysfunction; Disorder of the upper esophageal sphincter.
Dyspnea/cough/throat irritation	Dyspnea; Subglottic stenosis; Cough; Chronic throat clearing; Post‐nasal drip (PND); Asthma; Supraglottic mass.
Infectious/inflammatory	Acute laryngitis; Laryngitis (unspecified); Ulcerative laryngitis; Candida laryngitis; Laryngeal candidiasis; Viral pharyngitis.
Bilateral vocal fold paralysis	Bilateral Vocal Fold paralysis; Bilateral complete vocal fold paralysis
Cancer lesion	Laryngeal malignant neoplasm; Laryngeal cancer; Carcinoma in situ of vocal fold; Radiation adverse effect, sequela.

### 2.4 Patient‐Reported Outcome Measures

The VHI‐10 and RSI were administered as part of routine clinical intake. The VHI‐10 is a validated 10‐item questionnaire assessing the perception of one's voice handicap, with total scores ranging from 0 to 40. The RSI is a validated 9‐item instrument assessing the severity of laryngopharyngeal reflux–related symptoms, with total scores ranging from 0 to 45. Higher scores on both instruments indicate greater symptom burden.

### 2.5 Covariates

Sociodemographic and clinical variables were extracted from the electronic medical record. Covariates included age, sex, race, body mass index (BMI), insurance status, documented diagnoses of depression and/or anxiety, medical comorbidities (hypertension, coronary artery disease, diabetes), smoking status, and laryngological diagnosis at presentation. Race (White, Black, and other/unknown) and sex were included as self‐reported demographic variables.

### 2.6 Statistical Analysis

Descriptive statistics were calculated for the overall cohort and stratified by ADI quartile. Continuous variables were summarized using medians and interquartile ranges (IQRs), and categorical variables using frequencies and percentages. Statistical significance was assessed using Kruskal‐Wallis testing for continuous variables and *χ* ^2^ testing for categorical variables. A two‐sided *p* < 0.05 was considered statistically significant for descriptive statistics. Median VHI‐10 and RSI scores were compared across ADI quartiles using the Kruskal‐Wallis test due to non‐normal score distributions. When the global test was statistically significant, post hoc pairwise comparisons were performed using Wilcoxon rank‐sum tests with Bonferroni adjustment for multiple comparisons.

Multivariable linear regression models were constructed to evaluate the association between ADI (modeled as a continuous percentile variable) and VHI‐10 and RSI scores, adjusting for age, sex, race, BMI, insurance status, depression/anxiety, medical comorbidities, and laryngeal diagnosis. Regression coefficients (*β*) with 95% confidence intervals (CIs) were reported. Adjusted linear regression models were constructed for the overall cohort, the subset with a history of LPRD/GERD, and each of the six diagnosis subsets; therefore, since a patient could belong to three groups and we performed three tests, a Bonferroni adjusted *p*‐value of 0.017 was considered statistically significant for regression analyses. All analyses were performed using R, version 3.5.1 (R Foundation for Statistical Computing).

## 3 Results

A total of 1310 adult patients were included in the final analysis. The cohort demonstrated a broad distribution of neighborhood‐level socioeconomic status across ADI quartiles, with representation across the full national spectrum of deprivation. The median age of the cohort was 58.5 (IQR: 28.8–88.2), with a predominance of female patients (65.6%), consistent with typical laryngology referral patterns (Table 1). The most common laryngeal diagnosis on initial consultation was functional dysphonia (33.9%).

### 3.1 VHI‐10 and RSI by ADI Quartile

Patients residing in the least deprived neighborhoods (ADI Quartile 1) had significantly lower median VHI‐10 scores compared to patients residing in the most deprived neighborhoods (ADI Quartile 4) (median 10 vs. 14, *p* < 0.001). Similarly, RSI scores were significantly lower among patients in the least deprived quartile compared to those in the most deprived quartile (median 15 vs. 20, *p* < 0.001) (Table 1).

A stepwise increase in median symptom severity was observed across increasing ADI quartiles for both PROMs, demonstrating a graded relationship between neighborhood deprivation and subjective symptom burden at the time of presentation (Tables 3 and 4).

**TABLE 3:** Comparing reflux severity index scores across area deprivation index quartiles for the overall cohort and primary diagnosis subsets.

Subset	ADI quartile	Median (IQR) RSI score	Global *p* ^a^
Overall cohort (*n* = 1310)			< 0.001
	Q1, least deprived (national rankings: 2–44)	15 (8–22)	
	Q2 (national rankings: 45–65)	15.5 (8–23)	
	Q3 (national rankings: 66–81)	17 (9–25)	
	Q4, most deprived (national rankings: 82–100)	20 (11–29)^b^	
Subset with a prior diagnosis of LPRD/GERD (*n* = 173)			0.11
	Q1, least deprived (national rankings: 2–37)	17.5 (11.75–27)	
	Q2 (national rankings: 39–63)	20 (13–27.25)	
	Q3 (national rankings: 64–80)	21.5 (15.75–28.25)	
	Q4, most deprived (national rankings: 81–100)	24 (18–35)	
Subset with primary diagnosis = not laryngeal (*n* = 110)			0.56
	Q1, least deprived (national rankings: 14–46)	8 (3–14)	
	Q2 (national rankings: 48–64)	6.5 (1.25–18.75)	
	Q3 (national rankings: 65–80)	8 (2.75–14.25)	
	Q4, most deprived (national rankings: 81–100)	11 (5–20.5)	
Subset with primary diagnosis = functional (*n* = 444)			< 0.001
	Q1, least deprived (national rankings: 2–39)	13 (6–20)	
	Q2 (national rankings: 40–61)	14 (7–19)	
	Q3 (national rankings: 62–80)	19 (10–27.25)^c^	
	Q4, most deprived (national rankings: 81–100)	22 (9–30.75)^d^	
Subset with primary diagnosis = neurological (*n* = 180)			0.36
	Q1, least deprived (national rankings: 9–54)	19 (10–25)	
	Q2 (national rankings: 55–69)	17 (12.5–22)	
	Q3 (national rankings: 70–83)	18 (10–27)	
	Q4, most deprived (national rankings: 84–100)	20 (12–28)	
Subset with primary diagnosis = benign lesions (*n* = 345)			0.19
	Q1, least deprived (national rankings: 5–46)	14 (8–21.25)	
	Q2 (national rankings: 47–66)	13 (7–22)	
	Q3 (national rankings: 68–82)	17 (8.5–25)	
	Q4, most deprived (national rankings: 83–100)	16 (11–26)	
Subset with primary diagnosis = oropharyngeal/esophageal dysphagia (*n* = 58)			0.02
	Q1, least deprived (national rankings: 7–45)	13 (11.5–23)	
	Q2 (national rankings: 50–62)	23.5 (17.25–31.5)	
	Q3 (national rankings: 64–82)	17.5 (12–24.75)	
	Q4, most deprived (national rankings: 83–100)	31 (19.5–38.5)^e^	
Subset with primary diagnosis = dyspnea/cough/throat irritation (*n* = 143)			0.10
	Q1, least deprived (national rankings: 2–34)	20 (13–25)	
	Q2 (national rankings: 35–57)	20 (13–25)	
	Q3 (national rankings: 58–77)	18 (12.5–25)	
	Q4, most deprived (national rankings: 79–100)	25 (19–33)	

Abbreviations: ADI, area deprivation index; GERD, gastroesophageal reflux disease; LPRD, laryngopharyngeal reflux disease; RSI, reflux severity index; VHI‐10, voice handicap index‐10.

^a^ Global *p*‐value derived from Kruskal‐Wallis test comparing ADI quartiles.

^b^ Significantly different from Q1 (*p* < 0.001), Q2 (*p* < 0.001), and Q3 (*p* = 0.02) after Bonferroni correction.

^c^ Significantly different from Q1 (*p* = 0.005) and Q2 (*p* = 0.004) after Bonferroni correction.

^d^ Significantly different from Q1 (*p* = 0.001) and Q2 (*p* = 0.001) after Bonferroni correction.

^e^ Significantly different from Q1 (*p* = 0.03) after Bonferroni correction.

**TABLE 4:** Comparing voice handicap index‐10 scores across area deprivation index quartiles for the overall cohort and primary diagnosis subsets.

Subset	ADI quartile	Median (IQR) VHI‐10 score	Global *p* ^a^
Overall cohort (*n* = 1310)			0.0001
	Q1, least deprived (national rankings: 2–44)	10 (2–18)^b^	
	Q2 (national rankings: 45–65)	13 (5–23)	
	Q3 (national rankings: 66–81)	14 (3–23)	
	Q4, most deprived (national rankings: 82–100)	14 (4–25)	
Subset with a prior diagnosis of LPRD/GERD (*n* = 173)			0.07
	Q1, least deprived (national rankings: 2–37)	7 (1.75–13)	
	Q2 (national rankings: 39–63)	10 (2–14.5)	
	Q3 (national rankings: 64–80)	9.5 (2.75–16.25)	
	Q4, most deprived (national rankings: 81–100)	15 (4–24)	
Subset with primary diagnosis = not laryngeal (*n* = 110)			0.06
	Q1, least deprived (national rankings: 14–46)	0 (0–9)	
	Q2 (national rankings: 48–64)	0 (0–8.75)	
	Q3 (national rankings: 65–80)	0 (0–3.25)	
	Q4, most deprived (national rankings: 81–100)	5 (0–13)	
Subset with primary diagnosis = functional (*n* = 444)			0.12
	Q1, least deprived (national rankings: 2–39)	11 (4–16)	
	Q2 (national rankings: 40–61)	11 (5.25–17.75)	
	Q3 (national rankings: 62–80)	15 (5–21)	
	Q4, most deprived (national rankings: 81–100)	13 (6–23)	
Subset with primary diagnosis = neurological (*n* = 180)			0.97
	Q1, least deprived (national rankings: 9–54)	20 (12–32)	
	Q2 (national rankings: 55–69)	21 (13–29.5)	
	Q3 (national rankings: 70–83)	22 (12–27)	
	Q4, most deprived (national rankings: 84–100)	21 (13–30)	
Subset with primary diagnosis = benign lesions (*n* = 345)			0.56
	Q1, least deprived (national rankings: 5–46)	14 (9–22)	
	Q2 (national rankings: 47–66)	17 (9–24)	
	Q3 (national rankings: 68–82)	18 (10–25.5)	
	Q4, most deprived (national rankings: 83–100)	16 (7–26)	
Subset with primary diagnosis = oropharyngeal/esophageal dysphagia (*n* = 58)			< 0.001
	Q1, least deprived (national rankings: 7–45)	0 (0–1.5)^c^	
	Q2 (national rankings: 50–62)	14 (6–27.5)	
	Q3 (national rankings: 64–82)	7.5 (3–17.25)	
	Q4, most deprived (national rankings: 83–100)	13 (9.5–15.5)	
Subset with primary diagnosis = dyspnea/cough/throat irritation (*n* = 143)			0.90
	Q1, least deprived (national rankings: 2–34)	4 (0–9)	
	Q2 (national rankings: 35–57)	4 (0–9)	
	Q3 (national rankings: 58–77)	2 (0–12)	
	Q4, most deprived (national rankings: 79–100)	3 (0–15.75)	

Abbreviations: ADI, area deprivation index; GERD, gastroesophageal reflux disease; LPRD, laryngopharyngeal reflux disease; RSI, reflux severity index; VHI‐10, voice handicap index‐10.

^a^ Global *p*‐value derived from Kruskal‐Wallis test comparing ADI quartiles.

^b^ Significantly different from Q2 (*p* = 0.007), Q3 (*p* = 0.009), and Q4 (*p* = 0.0002) after Bonferroni correction.

^c^ Significantly different from Q2 (*p* = 0.006), Q3 (*p* = 0.04), and Q4 (*p* = 0.04) after Bonferroni correction.

### 3.2 Multivariable Associations Between ADI and PROMs

After adjustment for sociodemographic and clinical covariates, increasing ADI remained independently associated with higher RSI scores (*β* = 0.04 per ADI percentile increase; 95% CI: 0.01–0.07; *p* = 0.006) (Table 5). Similarly, increasing ADI was independently associated with higher VHI‐10 scores (*β* = 0.04 per ADI percentile increase; 95% CI: 0.008–0.06; *p* = 0.01) (Table 6).

**TABLE 5:** Linear regression examining the association between area deprivation index and reflux severity index score.

Subset	Variable	Unadjusted Beta (95% CI)	Unadjusted *p* ^a^	Adjusted beta^b^ (95% CI)	Adjusted *p* ^a^
Overall cohort (*n* = 1309)^c^	ADI national ranking value	0.07 (0.05–0.10)	< 0.001	0.04 (0.01, 0.07)	0.006
Subset with a prior diagnosis of LPRD/GERD (*n* = 173)	ADI national ranking value	0.09 (0.03–0.15)	0.004	0.06 (−0.02, 0.14)	0.14
Subset with primary diagnosis = not laryngeal (*n* = 110)	ADI national ranking value	0.06 (−0.03, 0.14)	0.22	0.04 (−0.07, 0.15)	0.49
Subset with primary diagnosis = functional (*n* = 443)^c^	ADI national ranking value	0.11 (0.07, 0.15)	< 0.001	0.06 (0.02, 0.11)	0.01
Subset with primary diagnosis = neurological (*n* = 180)	ADI national ranking value	0.07 (−0.01, 0.15)	0.11	0.04 (−0.06, 0.13)	0.47
Subset with primary diagnosis = benign (*n* = 345)	ADI national ranking value	0.05 (0.004, 0.09)	0.03	0.01 (−0.05, 0.06)	0.76
Subset with primary diagnosis = oropharyngeal/esophageal dysphagia (*n* = 58)	ADI national ranking value	0.15 (0.03, 0.27)	0.02	0.03 (−0.15, 0.21)	0.74
Subset with primary diagnosis = dyspnea/cough/throat irritation (*n* = 143)	ADI national ranking value	0.04 (−0.02, 0.11)	0.21	0.02 (−0.06, 0.11)	0.55

Abbreviations: ADI, area deprivation index; GERD, gastroesophageal reflux disease; LPRD, laryngopharyngeal reflux disease; RSI, reflux severity index; VHI‐10, voice handicap index‐10.

^a^ A Bonferroni adjusted *p*‐value of 0.017 was considered statistically significant.

^b^ Adjusted models for the overall cohort adjusted for ADI, insurance status, race, sex, every smoking status, age, BMI, hypertension, diabetes, coronary artery disease, depression/anxiety, and primary diagnosis category; adjusted models for the primary diagnosis subsets adjusted for ADI, insurance status, race, sex, every smoking status, age, BMI, hypertension, diabetes, coronary artery disease, and depression/anxiety.

^c^ One patient with unknown sex was excluded from these models.

**TABLE 6:** Linear regression examining the association between area deprivation index and voice handicap index‐10 score.

Subset	Variable	Unadjusted beta (95% CI)	Unadjusted *p* ^a^	Adjusted beta^b^ (95% CI)	Adjusted *p* ^a^
Overall cohort (*n* = 1309)^c^	ADI national ranking value	0.06 (0.04, 0.09)	< 0.001	0.04 (0.008, 0.06)	0.01
Subset with a prior diagnosis of LPRD/GERD (*n* = 173)	ADI national ranking value	0.09 (0.03, 0.15)	0.003	0.05 (−0.02, 0.12)	0.15
Subset with primary diagnosis = not laryngeal (*n* = 110)	ADI national ranking value	0.05 (−0.03, 0.13)	0.23	0.02 (−0.07, 0.10)	0.71
Subset with primary diagnosis = functional (*n* = 443)^c^	ADI national ranking value	0.05 (0.009, 0.09)	0.02	0.02 (−0.03, 0.06)	0.48
Subset with primary diagnosis = neurological (*n* = 180)	ADI national ranking value	0.03 (−0.06, 0.12)	0.48	0.07 (−0.04, 0.17)	0.2
Subset with primary diagnosis = benign (*n* = 345)	ADI national ranking value	0.03 (−0.02, 0.08)	0.21	0.04 (−0.02, 0.10)	0.15
Subset with primary diagnosis = oropharyngeal‐esophageal dysphagia (*n* = 58)	ADI national ranking value	0.15 (0.03, 0.28)	0.02	0.19 (−0.01, 0.38)	0.06
Subset with primary diagnosis = dyspnea/cough/throat irritation (*n* = 143)	ADI national ranking value	0.05 (−0.008, 0.11)	0.09	−0.01 (−0.07, 0.06)	0.77

Abbreviations: ADI, area deprivation index; GERD, gastroesophageal reflux disease; LPRD, laryngopharyngeal reflux disease; RSI, reflux severity index; VHI‐10, voice handicap index‐10.

^a^ A Bonferroni adjusted *p*‐value of 0.017 was considered statistically significant.

^b^ Adjusted models for the overall cohort adjusted for ADI, insurance status, race, sex, every smoking status, age, BMI, hypertension, diabetes, coronary artery disease, depression/anxiety, and primary diagnosis category; adjusted models for the primary diagnosis subsets adjusted for ADI, insurance status, race, sex, every smoking status, age, BMI, hypertension, diabetes, coronary artery disease, and depression/anxiety.

^c^ One patient with unknown sex was excluded from these models.

In the adjusted linear regression model for the overall cohort, other factors significantly associated with RSI scores were insurance status, sex, BMI, and primary diagnosis (Supporting Information S3). Primary diagnosis was the only variable besides ADI that was significantly associated with VHI‐10 scores for the overall cohort (Supporting Information S4).

When diagnostic subgroups were examined, no significant differences in VHI‐10 or RSI scores by ADI quartile were observed among patients whose primary diagnosis was non‐laryngeal. In contrast, among functional laryngeal disorders, RSI scores differed significantly among ADI quartile comparisons (Q1 vs. Q3, Q1 vs. Q4, Q2 vs. Q3, and Q2 vs. Q4), although VHI‐10 did not differ significantly. Consistent with these findings, regression models for the functional diagnosis subset demonstrated a significant association between ADI and RSI scores, both before and after adjustment for covariates, supporting a robust association between neighborhood deprivation and functional symptom burden.

Neurologic laryngeal disorders showed no significant association between ADI and PROMs, suggesting that symptom perception in this group may be less influenced by social context and stress physiology.

Among patients with benign structural pathology, ADI was not significantly associated with RSI or VHI‐10. Dysphagia‐related diagnosis demonstrated significant differences across ADI quartiles for both RSI and VHI‐10; however, no significant differences were observed among patients presenting with dyspnea, cough, or chronic throat clearing, which may in part reflect the smaller sample size of this subgroup (10.9%).

## 4 Discussion

In this large tertiary‐care laryngology cohort, higher neighborhood socioeconomic deprivation, as measured by the ADI, was independently associated with worse patient‐reported laryngeal symptoms. Patients living in the most deprived neighborhoods demonstrated significantly higher VHI‐10 and RSI scores at presentation compared with those in the least deprived quartile. These associations persisted after adjustment for demographic and clinical covariates. These findings suggest that neighborhood‐level disadvantage meaningfully impacts subjective voice and reflux symptom burden beyond individual‐level clinical factors.

Collectively, these findings support the concept that functional laryngeal disorders may be particularly sensitive to the influence of neighborhood‐level stress exposure and social context. However, disorders with clearer structural or neurologic etiologies may demonstrate weaker or inconsistent associations. This observation aligns with the proposed framework of allostatic load‐mediated laryngeal symptom amplification and helps contextualize the persistent disparities in RSI and VHI‐10 scores among patients from socioeconomically deprived neighborhoods.

Our study reveals differential findings between the VHI‐10 and RSI across diagnostic subgroups. This likely reflects the distinct constructs captured by these instruments. The VHI‐10 primarily measures the perceived impact of voice impairment in communication and quality of life, with items focused on vocal effort, voice reliability, and functional limitation during speech [6]. In contrast, the RSI captures a broader range of upper aerodigestive tract symptoms, including throat clearing, globus sensation, cough, dysphagia, and laryngopharyngeal irriation [7]. Because of this broader symptom scope, RSI may be more sensitive to symptom complexes extending beyond phonatory dysfunction alone. This difference in symptom domains may explain why RSI remained significant across ADI quartiles in multivariable models within certain diagnostic subgroups, whereas VHI‐10 differences were less consistent after adjustment. Patients may report persistent throat discomfort, globus or swallowing difficulty even in the absence of significant voice‐related handicap, leading to strong associations in RSI relative to the more voice‐specific VHI‐10.

The prominence of RSI differences within the functional laryngeal and dysphagia diagnostic categories further supports this interpretation. Functional laryngeal disorders are characterized as conditions in which voice or laryngeal symptoms arise from abnormal use or coordination of the laryngeal musculature despite gross normal laryngeal anatomy and intact motor function [21, 22].

In this cohort, functional laryngeal disorders included conditions such as muscle tension dysphonia (MTDa), inducible laryngeal obstruction (ILO), and irritable larynx (IL) which frequently present with overlapping symptoms of throat tightness, globus sensation, chronic throat clearing and cough in addition to voice changes [21, 22, 23]. Similarly, the dysphagia category encompasses a broad spectrum of dysphagia disorders that may be associated with heightened laryngopharyngeal sensory perception, muscle tension‐related swallowing dysfunction or symptoms attributed to reflux physiology [24]. It is important to note that often muscle tension dysphagia (MTDg) was not explicitly listed but may be included in this cohort under dysphagia unspecified since this study was performed prior to the inception of the International Classification of Diseases (ICD) 10 for MTDg (R13.10).

Most RSI queries directly capture these experiences such as globus, throat clearing, dysphagia, and throat irritation, whereas VHI‐10 focuses narrowly on vocal function. Consequently, RSI may better reflect the multisystem symptoms burden present in these groups, particularly when symptoms arise from overlapping laryngeal, sensory, or tension‐related mechanisms rather than isolated phonatory impairment. Although the precise etiologic drivers are not described in the present dataset, this pattern suggests that capturing broader aerodigestive symptomatology with the RSI may be more sensitive to contextual and physiologic influences associated with neighborhood deprivation, particularly among patients with functional laryngeal and swallowing‐related presentations. This pattern may be consistent with the proposed role of stress‐related burden in shaping symptom perception [25], making RSI scores potentially more sensitive than VHI‐10 to the physiologic and perceptual effects of this physiologic burden reflected by the ADI.

Prior otolaryngology literature has demonstrated that patients in high‐deprivation neighborhoods experience delayed diagnosis, reduced access to specialty care, and worse oncologic outcomes, particularly in head and neck cancer populations [12, 14, 26]. Similar disparities have been described in hearing healthcare access, pediatric airway disease, and chronic rhinosinusitis [13, 14, 27]. Our findings expand this work to outpatient laryngology and demonstrate that disparities are not only evident in access and survival outcomes but also in subjective symptom burden at the time of specialty evaluation. This suggests that patients living in high‐ADI neighborhoods may already carry a greater functional and symptomatic burden by the time they receive laryngological care.

Beyond access‐related barriers, emerging evidence indicates that socioeconomic deprivation exerts direct physiologic effects through chronic stress‐mediated biologic dysregulation, termed allostatic load [8, 9]. Allostatic load reflects cumulative activation of the hypothalamic–pituitary–adrenal axis, autonomic nervous system, immune signaling, and cardiometabolic pathways in response to chronic stress exposure. Elevated allostatic load has been associated with increased morbidity, depression, chronic inflammation, and premature mortality across a wide range of pathology [17, 18, 28]. Neighborhood deprivation is an influential upstream contributor to cumulative biological burden. The ADI has been linked to increased inflammatory biomarkers, dysregulated cortisol patterns, adverse cardiometabolic profiles, and reduced heart rate variability (HRV) [11, 13, 14]. Together, these data suggest that ADI captures not only structural deprivation but also stress‐mediated physiologic vulnerability that may directly influence how symptoms are perceived, processed, and reported.

The minimal clinically important difference (MCID) is an assessment tool that characterizes the smallest change in an outcome that is meaningful to patients, thereby bridging the gap between statistical analysis and clinical relevance. Studies evaluating clinically meaningful change have estimated the MCID for the VHI‐10 to be approximately 5 to 6 points, representing the smallest difference perceived by patients as clinically important [29, 30]. In our study, the uncorrected VHI‐10 scores, reflecting patient‐reported voice handicap at initial presentation prior to any therapeutic intervention, demonstrate a difference between patients residing in the least deprived and most deprived ADI quartiles that met or exceeded this threshold. Although adjusted analyses attenuated some subgroup differences, the magnitude of the baseline disparity suggests that patients from more socioeconomically deprived neighborhoods may present with clinically meaningful increases in perceived voice handicap even before treatment is initiated. It should be noted, however, that application of the MCID in this context remains speculative, as MCID thresholds for the VHI‐10 have largely been derived using anchor‐based methods evaluating within‐patient changes before and after therapeutic interventions, rather than differences between population groups. Nonetheless, there is literature that addresses that variations in MCID have been observed across different patient demographics and thus cross‐cultural studies are needed to validate MCID in diverse populations [31].

In the field of otolaryngology, disparities in patient‐reported outcomes have been described in chronic rhinosinusitis, postoperative otolaryngology outcomes, and head and neck cancer survivorship. Lower socioeconomic status and minority race have been associated with worse baseline PROMs scores and differential response to therapy [32, 33, 34]. Our study is among the first to demonstrate this phenomenon specifically within a general laryngology population using validated voice‐ and reflux‐specific PROMs in conjunction with ADI. This study, while based on geographic deprivation alone, helps to lay the groundwork for further mechanistic studies by demonstrating that patients from more deprived neighborhoods present to specialty care with a higher subjective symptom burden.

The observed association between ADI and PROMs has several practical implications. First, VHI‐10 and RSI scores are frequently used to triage referrals, guide shared decision‐making, and monitor treatment response. Unrecognized influences of neighborhood deprivation and stress biology on these scores may lead to under‐ or over‐estimation of disease severity if interpreted in isolation from social context. Second, identifying patients from high‐deprivation neighborhoods as being at risk for greater symptom burden may help clinicians prioritize early evaluation, care coordination, and multidisciplinary support (including speech‐language pathology and behavioral health) for these individuals.

At a systems level, our findings support the need for equity‐focused quality improvement efforts in laryngology that incorporate neighborhood‐level metrics such as ADI. Similar to work in head and neck oncology showing that patients from high‐ADI neighborhoods experience delays in adjuvant therapy and worse survivorship outcomes [35, 36], laryngology practices may consider using ADI mapping to identify catchment areas with high deprivation, guide community outreach, and design telehealth or navigation interventions to reduce barriers to timely specialty care.

This study has several limitations. First, its retrospective, cross‐sectional design precludes causal inference. While we observed robust associations between ADI and VHI‐10/RSI scores at presentation, we cannot determine temporality or definitively establish that neighborhood deprivation causes greater symptom burden. Second, ADI is an area‐level measure that may not capture within‐neighborhood heterogeneity or individual‐level socioeconomic factors such as income, education, or employment; thus, residual confounding by unmeasured individual‐level socioeconomic factors is possible. Third, although we adjusted for key clinical and sociodemographic variables, additional factors, such as symptom duration, prior treatment history, health literacy, perceived discrimination, social support, and detailed mental health symptom burden were not assessed and may influence both ADI and PROMs.

Our findings highlight several priorities for future research. Prospective, longitudinal studies are necessary to assess how ADI and allostatic load jointly influence laryngeal symptom trajectories and treatment responses over time. These studies should include validated allostatic load indices, such as inflammatory markers, metabolic parameters, and cardiovascular measures, alongside PROMs and objective laryngeal assessments (acoustic measures, aerodynamic testing, and laryngoscopic findings). Disease‐specific investigations will also be essential. For instance, muscle tension dysphonia/dysphagia, benign phonotraumatic lesions, and laryngopharyngeal reflux may be particularly affected by chronic stress and psychosocial factors, making them promising targets for combined ADI‐allostatic load studies. Incorporating neighborhood‐level data with detailed clinical phenotyping could help determine whether higher deprivation and physiological stress correlate with distinct patterns of laryngeal pathology (e.g., hyperfunctional patterns, increased pain or pressure ratings, or discrepancies between objective and subjective findings).

## 5 Conclusion

In summary, neighborhood‐level socioeconomic deprivation, as measured by ADI, is independently associated with worse VHI‐10 and RSI scores at presentation to a tertiary‐care laryngology clinic. These findings suggest that patients living in more deprived neighborhoods may experience both structural barriers to care and heightened physiologic stress burden that together amplify subjective laryngeal symptomatology. Incorporating neighborhood context and allostatic load into laryngology research and clinical practice will be essential to developing targeted, multi‐level interventions that advance equity in laryngology care.

## Funding

The authors have nothing to report.

## Conflicts of Interest

The authors declare no conflicts of interest.

## Supporting information


**Data S1:** CONSORT diagram of study sample.
**Supporting Information: S2** Primary diagnosis groupings of non‐laryngeal disease.
**Supporting Information: S3** Linear regression examining factors associated with Reflux Severity Index score.
**Supporting Information: S4** Linear regression examining factors associated with Voice Handicap Index‐10 score.

## Data Availability

The data that support the findings of this study are available from the corresponding author upon reasonable request.
