# Supplementary material for: Neighborhood Deprivation and Voice and Reflux Symptom Burden in a Tertiary Laryngology Cohort
Source: Laryngoscope. 2026 May 6;136(9):3826–40. doi: 10.1002/lary.70610 (PMC13460870; doi:10.1002/lary.70610)
Supplement: Supplementary file 1 [file LARY-136-3826-s001.docx]

Supplementary Material

**Supplementary Material 1**. CONSORT diagram of study sample

**Supplementary Material 2**. Primary diagnosis groupings of non-laryngeal disease

**Supplementary Material 3**. Linear regression examining factors associated with Reflux Severity Index score

**Supplementary Material 4**. Linear regression examining factors associated with Voice Handicap Index-10 score

**Supplementary Material 1**. CONSORT diagram of study sample

**Supplementary Material 2**. Non-Laryngeal Diagnoses

| **Diagnosis category** | **Condition** |
| --- | --- |
| Non-laryngeal | Abdominal aortic aneurysm, ruptured; Abnormal brain imaging; Acquired absence of other organs; Acute postprocedural aspiration pneumonitis; Acute postprocedural respiratory failure; Anosmia; Benign neoplasm of unspecified soft tissue; Benign neoplasm, unspecified; Childhood onset fluency disorder; Conversion disorder with motor symptom; Encounter for other preprocedural examination; Epistaxis; General adult medical examination without abnormal findings; Hemoptysis; Impacted cerumen, bilateral; Intestinal bypass and anastomosis status; Multiple myeloma not having achieved remission; Neoplasm of uncertain behavior of aorta; Neoplasm of uncertain behavior of other endocrine glands; Noncompliance with medical treatment; Obstructive sleep apnea (adult and pediatric); Other specified health status; Other specified injuries of head, initial encounter; Other specified symptoms and signs involving the circulatory or respiratory systems; Otalgia, left ear; Otalgia, right ear; Otitis media, unspecified, bilateral; Parosmia; Personal history of immunosuppressive drug therapy; Personal history of other diseases of the nervous system; Postprocedural status, other; Presence of aortocoronary bypass graft; Sensorineural hearing loss, bilateral; Temporomandibular joint disorder, unspecified; Toxic effect of unspecified drug, subsequent encounter; Tracheobronchitis, not specified as acute or chronic; Unspecified asthma, uncomplicated; Unspecified intellectual disabilities; Unspecified sensorineural hearing loss. |

**Supplementary Material 3**. Linear regression examining factors associated with Reflux Severity Index score

| **Subset** | **Variable** | **Unadjusted Beta (95% CI)** | **Unadjusted p-value*** | **Adjusted Beta (95% CI)** | **Adjusted p-value*** |
| --- | --- | --- | --- | --- | --- |
| **Overall cohort (n=1309)**** |  |  |  |  |  |
|  | ADI national ranking value | 0.07 (0.05-0.10) | <0.001 | 0.04 (0.01, 0.07) | 0.006 |
|  | Insurance status: private/managed care (ref) |  |  | 1 |  |
|  | Insurance status: Medicaid |  |  | 2.90 (0.85, 4.95) | 0.006 |
|  | Insurance status: Medicare |  |  | 0.73 (-1.05, 2.50) | 0.42 |
|  | Insurance status: other/unknown |  |  | 5.24 (1.29, 9.19) | 0.01 |
|  | Race: White (ref) |  |  | 1 |  |
|  | Race: Black |  |  | 1.76 (-0.39, 3.92) | 0.11 |
|  | Race: other/unknown |  |  | -1.93 (-4.48, 0.62) | 0.14 |
|  | Sex: female (ref) |  |  | 1 |  |
|  | Sex: male |  |  | -2.39 (-3.73, -1.06) | <0.001 |
|  | Ever smoking status: no (ref) |  |  | 1 |  |
|  | Ever smoking status: yes |  |  | 1.59 (0.27, 2.91) | 0.02 |
|  | Age |  |  | 0.02 (-0.03, 0.07) | 0.41 |
|  | BMI |  |  | 0.12 (0.03, 0.21) | 0.01 |
|  | Hypertension: no (ref) |  |  | 1 |  |
|  | Hypertension: yes |  |  | 0.95 (-0.57, 2.46) | 0.22 |
|  | Diabetes: no (ref) |  |  | 1 |  |
|  | Diabetes: yes |  |  | -0.81 (-2.87, 1.26) | 0.44 |
|  | Coronary artery disease: no (ref) |  |  | 1 |  |
|  | Coronary artery disease: yes |  |  | 0.46 (-2.91, 3.83) | 0.79 |
|  | Depression/anxiety: no (ref) |  |  | 1 |  |
|  | Depression/anxiety: yes |  |  | 1.64 (0.16, 3.12) | 0.03 |
|  | Primary diagnosis: not laryngeal (ref) |  |  | 1 |  |
|  | Primary diagnosis: benign |  |  | 4.57 (2.17, 6.98) | <0.001 |
|  | Primary diagnosis: dyspnea/cough/throat irritation |  |  | 8.76 (5.95, 11.58) | <0.001 |
|  | Primary diagnosis: functional |  |  | 5.93 (3.60, 8.26) | <0.001 |
|  | Primary diagnosis: neurological |  |  | 6.99 (4.35, 9.62) | <0.001 |
|  | Primary diagnosis: oropharyngoesophageal/dysphagia |  |  | 10.53 (6.76, 14.31) | <0.001 |
|  | Primary diagnosis: other** |  |  | 11.11 (6.49, 15.73) | <0.001 |
|  |  |  |  |  |  |
| **Subset with a prior diagnosis of LPRD/GERD (n=173)** |  |  |  |  |  |
|  | ADI national ranking value | 0.09 (0.03-0.15) | 0.004 | 0.06 (-0.02, 0.14) | 0.14 |
|  | Insurance status: private/managed care (ref) |  |  | 1 |  |
|  | Insurance status: Medicaid |  |  | 4.42 (-0.71, 9.55) | 0.09 |
|  | Insurance status: Medicare |  |  | 1.68 (-3.59, 6.95) | 0.53 |
|  | Insurance status: other/unknown |  |  | 7.07 (-1.03, 15.17) | 0.09 |
|  | Race: White (ref) |  |  | 1 |  |
|  | Race: Black |  |  | 1.53 (-4.28, 7.33) | 0.6 |
|  | Race: other/unknown |  |  | 0.21 (-5.95, 6.37) | 0.95 |
|  | Sex: female (ref) |  |  | 1 |  |
|  | Sex: male |  |  | 0.64 (-3.07, 4.36) | 0.73 |
|  | Ever smoking status: no (ref) |  |  | 1 |  |
|  | Ever smoking status: yes |  |  | 1.83 (-1.79, 5.44) | 0.32 |
|  | Age |  |  | -0.04 (-0.17, 0.09) | 0.56 |
|  | BMI |  |  | 0.01 (-0.23, 0.25) | 0.92 |
|  | Hypertension: no (ref) |  |  | 1 |  |
|  | Hypertension: yes |  |  | 3.37 (-1.50, 8.25) | 0.17 |
|  | Diabetes: no (ref) |  |  | 1 |  |
|  | Diabetes: yes |  |  | 6.98 (0.52, 13.43) | 0.03 |
|  | Coronary artery disease: no (ref) |  |  | 1 |  |
|  | Coronary artery disease: yes |  |  | -14.58 (-25.98, -3.18) | 0.01 |
|  | Depression/anxiety: no (ref) |  |  | 1 |  |
|  | Depression/anxiety: yes |  |  | 2.05 (-1.74, 5.84) | 0.29 |
|  |  |  |  |  |  |
| **Subset with primary diagnosis = not laryngeal (n=110**) |  |  |  |  |  |
|  | ADI national ranking value | 0.06 (-0.03, 0.14) | 0.22 | 0.04 (-0.07, 0.15) | 0.49 |
|  | Insurance status: private/managed care (ref) |  |  | 1 |  |
|  | Insurance status: Medicaid |  |  | 4.34 (-2.23, 10.92) | 0.19 |
|  | Insurance status: Medicare |  |  | 1.28 (-4.70, 7.25) | 0.67 |
|  | Insurance status: other/unknown |  |  | -14.29 (-36.28, 7.70) | 0.2 |
|  | Race: White (ref) |  |  | 1 |  |
|  | Race: Black |  |  | 8.67 (-0.16, 17.50) | 0.054 |
|  | Race: other/unknown |  |  | 0.71 (-8.24, 9.66) | 0.87 |
|  | Sex: female (ref) |  |  | 1 |  |
|  | Sex: male |  |  | 1.41 (-3.12, 5.93) | 0.54 |
|  | Ever smoking status: no (ref) |  |  | 1 |  |
|  | Ever smoking status: yes |  |  | -2.81 (-7.44, 1.82) | 0.23 |
|  | Age |  |  | -0.02 (-0.20, 0.16) | 0.82 |
|  | BMI |  |  | 0.21 (-0.10, 0.53) | 0.18 |
|  | Hypertension: no (ref) |  |  | 1 |  |
|  | Hypertension: yes |  |  | 2.86 (-2.41, 8.13) | 0.28 |
|  | Diabetes: no (ref) |  |  | 1 |  |
|  | Diabetes: yes |  |  | -2.22 (-11.01, 6.57) | 0.62 |
|  | Coronary artery disease: no (ref) |  |  | 1 |  |
|  | Coronary artery disease: yes |  |  | -9.16 (-25.40, 7.08) | 0.27 |
|  | Depression/anxiety: no (ref) |  |  | 1 |  |
|  | Depression/anxiety: yes |  |  | -0.41 (-5.52, 4.69) | 0.87 |
|  |  |  |  |  |  |
| **Subset with primary diagnosis = functional (n=443)**** |  |  |  |  |  |
|  | ADI national ranking value | 0.11 (0.07, 0.15) | <0.001 | 0.06 (0.02, 0.11) | 0.01 |
|  | Insurance status: private/managed care (ref) |  |  | 1 |  |
|  | Insurance status: Medicaid |  |  | 3.16 (-0.35, 6.66) | 0.08 |
|  | Insurance status: Medicare |  |  | -0.34 (-3.59, 2.91) | 0.84 |
|  | Insurance status: other/unknown |  |  | 4.66 (-1.62, 10.94) | 0.15 |
|  | Race: White (ref) |  |  | 1 |  |
|  | Race: Black |  |  | -1.38 (-5.24, 2.47) | 0.48 |
|  | Race: other/unknown |  |  | -2.62 (-6.73, 1.48) | 0.21 |
|  | Sex: female (ref) |  |  | 1 |  |
|  | Sex: male |  |  | -3.48 (-6.00, -0.97) | 0.01 |
|  | Ever smoking status: no (ref) |  |  | 1 |  |
|  | Ever smoking status: yes |  |  | 0.83 (-1.57, 3.23) | 0.5 |
|  | Age |  |  | 0.09 (0.00, 0.17) | 0.04 |
|  | BMI |  |  | 0.18 (0.03, 0.34) | 0.02 |
|  | Hypertension: no (ref) |  |  | 1 |  |
|  | Hypertension: yes |  |  | 0.96 (-2.13, 4.05) | 0.54 |
|  | Diabetes: no (ref) |  |  | 1 |  |
|  | Diabetes: yes |  |  | -0.26 (-4.07, 3.56) | 0.89 |
|  | Coronary artery disease: no (ref) |  |  | 1 |  |
|  | Coronary artery disease: yes |  |  | -2.61 (-10.46, 5.23) | 0.51 |
|  | Depression/anxiety: no (ref) |  |  | 1 |  |
|  | Depression/anxiety: yes |  |  | 0.81 (-1.69, 3.31) | 0.52 |
|  |  |  |  |  |  |
| **Subset with primary diagnosis = neurological (n=180)** |  |  |  |  |  |
|  | ADI national ranking value | 0.07 (-0.01, 0.15) | 0.11 | 0.04 (-0.06, 0.13) | 0.47 |
|  | Insurance status: private/managed care (ref) |  |  | 1 |  |
|  | Insurance status: Medicaid |  |  | 0.24 (-6.11, 6.59) | 0.94 |
|  | Insurance status: Medicare |  |  | 1.36 (-3.50, 6.23) | 0.58 |
|  | Insurance status: other/unknown |  |  | 11.26 (0.64, 21.89) | 0.04 |
|  | Race: White (ref) |  |  | 1 |  |
|  | Race: Black |  |  | 2.57 (-3.72, 8.86) | 0.42 |
|  | Race: other/unknown |  |  | 5.29 (-2.66, 13.24) | 0.19 |
|  | Sex: female (ref) |  |  | 1 |  |
|  | Sex: male |  |  | -2.96 (-6.71, 0.79) | 0.12 |
|  | Ever smoking status: no (ref) |  |  | 1 |  |
|  | Ever smoking status: yes |  |  | 3.95 (0.22, 7.68) | 0.04 |
|  | Age |  |  | -0.03 (-0.18, 0.13) | 0.74 |
|  | BMI |  |  | -0.13 (-0.41, 0.14) | 0.34 |
|  | Hypertension: no (ref) |  |  | 1 |  |
|  | Hypertension: yes |  |  | 0.83 (-3.24, 4.90) | 0.69 |
|  | Diabetes: no (ref) |  |  | 1 |  |
|  | Diabetes: yes |  |  | -1.98 (-7.34, 3.37) | 0.47 |
|  | Coronary artery disease: no (ref) |  |  | 1 |  |
|  | Coronary artery disease: yes |  |  | -4.93 (-12.30, 2.43) | 0.19 |
|  | Depression/anxiety: no (ref) |  |  | 1 |  |
|  | Depression/anxiety: yes |  |  | -1.30 (-5.61, 3.01) | 0.55 |
|  |  |  |  |  |  |
| **Subset with primary diagnosis = benign (n=345)** |  |  |  |  |  |
|  | ADI national ranking value | 0.05 (0.004, 0.09) | 0.03 | 0.01 (-0.05, 0.06) | 0.76 |
|  | Insurance status: private/managed care (ref) |  |  | 1 |  |
|  | Insurance status: Medicaid |  |  | 4.47 (0.36, 8.57) | 0.03 |
|  | Insurance status: Medicare |  |  | 2.42 (-1.19, 6.03) | 0.19 |
|  | Insurance status: other/unknown |  |  | 6.13 (-0.92, 13.18) | 0.09 |
|  | Race: White (ref) |  |  | 1 |  |
|  | Race: Black |  |  | 0.60 (-3.22, 4.42) | 0.76 |
|  | Race: other/unknown |  |  | -2.47 (-9.19, 4.26) | 0.47 |
|  | Sex: female (ref) |  |  | 1 |  |
|  | Sex: male |  |  | -0.70 (-3.21, 1.82) | 0.59 |
|  | Ever smoking status: no (ref) |  |  | 1 |  |
|  | Ever smoking status: yes |  |  | 3.14 (0.69, 5.59) | 0.01 |
|  | Age |  |  | -0.05 (-0.15, 0.05) | 0.35 |
|  | BMI |  |  | 0.10 (-0.08, 0.27) | 0.28 |
|  | Hypertension: no (ref) |  |  | 1 |  |
|  | Hypertension: yes |  |  | 1.44 (-1.22, 4.10) | 0.29 |
|  | Diabetes: no (ref) |  |  | 1 |  |
|  | Diabetes: yes |  |  | 0.19 (-3.86, 4.24) | 0.93 |
|  | Coronary artery disease: no (ref) |  |  | 1 |  |
|  | Coronary artery disease: yes |  |  | 4.10 (-1.53, 9.73) | 0.15 |
|  | Depression/anxiety: no (ref) |  |  | 1 |  |
|  | Depression/anxiety: yes |  |  | 2.29 (-0.60, 5.18) | 0.12 |
|  |  |  |  |  |  |
| **Subset with primary diagnosis = oropharyngoesophageal or dysphagia (n=58)** |  |  |  |  |  |
|  | ADI national ranking value | 0.15 (0.03, 0.27) | 0.02 | 0.03 (-0.15, 0.21) | 0.74 |
|  | Insurance status: private/managed care (ref) |  |  | 1 |  |
|  | Insurance status: Medicaid |  |  | 2.76 (-9.02, 14.54) | 0.64 |
|  | Insurance status: Medicare |  |  | 8.73 (-1.67, 19.12) | 0.1 |
|  | Insurance status: other/unknown |  |  | 6.08 (-18.24, 30.39) | 0.61 |
|  | Race: White (ref) |  |  | 1 |  |
|  | Race: Black |  |  | 11.47 (-1.09, 24.03) | 0.07 |
|  | Race: other/unknown |  |  | -2.18 (-15.62, 11.27) | 0.74 |
|  | Sex: female (ref) |  |  | 1 |  |
|  | Sex: male |  |  | -4.88 (-13.20, 3.44) | 0.24 |
|  | Ever smoking status: no (ref) |  |  | 1 |  |
|  | Ever smoking status: yes |  |  | 1.18 (-6.47, 8.83) | 0.75 |
|  | Age |  |  | -0.14 (-0.39, 0.12) | 0.28 |
|  | BMI |  |  | 0.28 (-0.37, 0.93) | 0.39 |
|  | Hypertension: no (ref) |  |  | 1 |  |
|  | Hypertension: yes |  |  | 1.59 (-7.27, 10.46) | 0.72 |
|  | Diabetes: no (ref) |  |  | 1 |  |
|  | Diabetes: yes |  |  | -4.43 (-14.97, 6.12) | 0.4 |
|  | Coronary artery disease: no (ref) |  |  | 1 |  |
|  | Coronary artery disease: yes |  |  | 9.73 (-6.26, 25.72) | 0.22 |
|  | Depression/anxiety: no (ref) |  |  | 1 |  |
|  | Depression/anxiety: yes |  |  | 11.73 (3.41, 20.05) | 0.007 |
|  |  |  |  |  |  |
| **Subset with primary diagnosis = dyspnea/cough/throat irritation (n=143)** |  |  |  |  |  |
|  | ADI national ranking value | 0.04 (-0.02, 0.11) | 0.21 | 0.02 (-0.06, 0.11) | 0.55 |
|  | Insurance status: private/managed care (ref) |  |  | 1 |  |
|  | Insurance status: Medicaid |  |  | 2.13 (-4.77, 9.03) | 0.54 |
|  | Insurance status: Medicare |  |  | -1.02 (-6.48, 4.44) | 0.71 |
|  | Insurance status: other/unknown |  |  | 19.29 (-0.97, 39.54) | 0.06 |
|  | Race: White (ref) |  |  | 1 |  |
|  | Race: Black |  |  | 6.41 (-0.41, 13.23) | 0.07 |
|  | Race: other/unknown |  |  | -1.03 (-7.63, 5.57) | 0.76 |
|  | Sex: female (ref) |  |  | 1 |  |
|  | Sex: male |  |  | -1.88 (-6.15, 2.40) | 0.39 |
|  | Ever smoking status: no (ref) |  |  | 1 |  |
|  | Ever smoking status: yes |  |  | 0.49 (-3.65, 4.63) | 0.82 |
|  | Age |  |  | 0.12 (-0.07, 0.30) | 0.21 |
|  | BMI |  |  | -0.01 (-0.33, 0.31) | 0.94 |
|  | Hypertension: no (ref) |  |  | 1 |  |
|  | Hypertension: yes |  |  | 0.88 (-4.01, 5.78) | 0.72 |
|  | Diabetes: no (ref) |  |  | 1 |  |
|  | Diabetes: yes |  |  | -7.76 (-14.59, -0.93) | 0.03 |
|  | Coronary artery disease: no (ref) |  |  | 1 |  |
|  | Coronary artery disease: yes |  |  | 2.99 (-9.27, 15.25) | 0.63 |
|  | Depression/anxiety: no (ref) |  |  | 1 |  |
|  | Depression/anxiety: yes |  |  | 4.73 (-0.38, 9.85) | 0.07 |

* A Bonferroni adjusted p-value of 0.017 was considered statistically significant

** One patient with unknown sex was excluded from these models

*** Includes primary diagnosis categories: cancer, infectious/inflammatory, and bilateral vocal fold paralysis

ADI: Area Deprivation Index; BMI: Body Mass Index; RSI: Reflux Severity Index; VHI-10: Voice Handicap Index-10; LPRD: Laryngopharyngeal Reflux Disease; GERD: Gastroesophageal Reflux Disease

**Supplementary Material 4**. Linear regression examining factors associated with Voice Handicap Index-10 score

| **Subset** | **Variable** | **Unadjusted Beta (95% CI)** | **Unadjusted p-value*** | **Adjusted Beta (95% CI)** | **Adjusted p-value*** |
| --- | --- | --- | --- | --- | --- |
| **Overall cohort (n=1309)**** |  |  |  |  |  |
|  | ADI national ranking value | 0.06 (0.04, 0.09) | <0.001 | 0.04 (0.008, 0.06) | 0.01 |
|  | Insurance status: private/managed care (ref) |  |  | 1 |  |
|  | Insurance status: Medicaid |  |  | 2.28 (0.23, 4.33) | 0.03 |
|  | Insurance status: Medicare |  |  | 0.45 (-1.33, 2.23) | 0.62 |
|  | Insurance status: other/unknown |  |  | -3.41 (-7.37, 0.54) | 0.09 |
|  | Race: White (ref) |  |  | 1 |  |
|  | Race: Black |  |  | 1.1 (-1.05, 3.26) | 0.32 |
|  | Race: other/unknown |  |  | 1 (-1.55, 3.56) | 0.44 |
|  | Sex: female (ref) |  |  | 1 |  |
|  | Sex: male |  |  | -0.06 (-1.40, 1.27) | 0.93 |
|  | Ever smoking status: no (ref) |  |  | 1 |  |
|  | Ever smoking status: yes |  |  | 0.13 | 0.85 |
|  | Age |  |  | -0.02 (-0.07, 0.03) | 0.48 |
|  | BMI |  |  | -0.03 (-0.12, 0.07) | 0.59 |
|  | Hypertension: no (ref) |  |  | 1 |  |
|  | Hypertension: yes |  |  | 0.51 (-1.0, 2.03) | 0.51 |
|  | Diabetes: no (ref) |  |  | 1 |  |
|  | Diabetes: yes |  |  | 0.03 (-2.04, 2.09) | 0.98 |
|  | Coronary artery disease: no (ref) |  |  | 1 |  |
|  | Coronary artery disease: yes |  |  | -0.5 (-3.87, 2.87) | 0.77 |
|  | Depression/anxiety: no (ref) |  |  | 1 |  |
|  | Depression/anxiety: yes |  |  | 1.78 (0.30, 3.26) | 0.02 |
|  | Primary diagnosis: not laryngeal (ref) |  |  | 1 |  |
|  | Primary diagnosis: benign |  |  | 10.53 (8.12, 12.94) | <0.001 |
|  | Primary diagnosis: dyspnea/cough/throat irritation |  |  | 1.24 (-1.58, 4.05) | 0.39 |
|  | Primary diagnosis: functional |  |  | 7.88 (5.55, 10.21) | <0.001 |
|  | Primary diagnosis: neurological |  |  | 15.23 (12.59, 17.87) | <0.001 |
|  | Primary diagnosis: oropharyngoesophageal/dysphagia |  |  | 4.81 (1.04, 8.59) | 0.01 |
|  | Primary diagnosis: other** |  |  | 14.77 (10.15, 19.39) | <0.001 |
|  |  |  |  |  |  |
| **Subset with a prior diagnosis of LPRD/GERD (n=173)** |  |  |  |  |  |
|  | ADI national ranking value | 0.09 (0.03, 0.15) | 0.003 | 0.05 (-0.02, 0.12) | 0.15 |
|  | Insurance status: private/managed care (ref) |  |  | 1 |  |
|  | Insurance status: Medicaid |  |  | 1.50 (-3.30, 6.30) | 0.54 |
|  | Insurance status: Medicare |  |  | -1.34 (-6.27, 3.59) | 0.59 |
|  | Insurance status: other/unknown |  |  | -0.32 (-7.90, 7.26) | 0.93 |
|  | Race: White (ref) |  |  | 1 |  |
|  | Race: Black |  |  | 0.58 (-4.85, 6.01) | 0.83 |
|  | Race: other/unknown |  |  | -0.69 (-6.45, 5.08) | 0.81 |
|  | Sex: female (ref) |  |  | 1 |  |
|  | Sex: male |  |  | 1.36 (-2.12, 4.83) | 0.44 |
|  | Ever smoking status: no (ref) |  |  | 1 |  |
|  | Ever smoking status: yes |  |  | 0.59 (-2.80, 3.97) | 0.73 |
|  | Age |  |  | 0.07 (-0.06, 0.19) | 0.31 |
|  | BMI |  |  | 0.07 (-0.15, 0.30) | 0.53 |
|  | Hypertension: no (ref) |  |  | 1 |  |
|  | Hypertension: yes |  |  | 0.68 (-3.88, 5.24) | 0.77 |
|  | Diabetes: no (ref) |  |  | 1 |  |
|  | Diabetes: yes |  |  | 6.15 (0.11, 12.19) | 0.05 |
|  | Coronary artery disease: no (ref) |  |  | 1 |  |
|  | Coronary artery disease: yes |  |  | -1.61 (-12.28, 9.05) | 0.77 |
|  | Depression/anxiety: no (ref) |  |  | 1 |  |
|  | Depression/anxiety: yes |  |  | 4.16 (0.61, 7.70) | 0.02 |
|  |  |  |  |  |  |
| **Subset with primary diagnosis = not laryngeal (n=110**) |  |  |  |  |  |
|  | ADI national ranking value | 0.05 (-0.03, 0.13) | 0.23 | 0.02 (-0.07, 0.10) | 0.71 |
|  | Insurance status: private/managed care (ref) |  |  | 1 |  |
|  | Insurance status: Medicaid |  |  | 6.87 (1.60, 12.13) | 0.01 |
|  | Insurance status: Medicare |  |  | -0.17 (-4.95, 4.61) | 0.94 |
|  | Insurance status: other/unknown |  |  | -4.37 (-21.96, 13.22) | 0.62 |
|  | Race: White (ref) |  |  | 1 |  |
|  | Race: Black |  |  | 9.21 (2.14, 16.28) | 0.01 |
|  | Race: other/unknown |  |  | -7.07 (-14.23, 0.09) | 0.053 |
|  | Sex: female (ref) |  |  | 1 |  |
|  | Sex: male |  |  | 2.19 (-1.43, 5.81) | 0.23 |
|  | Ever smoking status: no (ref) |  |  | 1 |  |
|  | Ever smoking status: yes |  |  | -3.29 (-7.00, 0.41) | 0.08 |
|  | Age |  |  | -0.06 (-0.21, 0.08) | 0.38 |
|  | BMI |  |  | -0.20 (-0.45, 0.05) | 0.12 |
|  | Hypertension: no (ref) |  |  | 1 |  |
|  | Hypertension: yes |  |  | 0.73 (-3.49, 4.95) | 0.73 |
|  | Diabetes: no (ref) |  |  | 1 |  |
|  | Diabetes: yes |  |  | 4.24 (-2.79, 11.27) | 0.23 |
|  | Coronary artery disease: no (ref) |  |  | 1 |  |
|  | Coronary artery disease: yes |  |  | -2.05 (-15.04, 10.94) | 0.75 |
|  | Depression/anxiety: no (ref) |  |  | 1 |  |
|  | Depression/anxiety: yes |  |  | -0.94 (-5.03, 3.14) | 0.65 |
|  |  |  |  |  |  |
| **Subset with primary diagnosis = functional (n=443)**** |  |  |  |  |  |
|  | ADI national ranking value | 0.05 (0.009, 0.09) | 0.02 | 0.02 (-0.03, 0.06) | 0.48 |
|  | Insurance status: private/managed care (ref) |  |  | 1 |  |
|  | Insurance status: Medicaid |  |  | 4.13 (0.70, 7.57) | 0.02 |
|  | Insurance status: Medicare |  |  | 2.34 (-0.84, 5.53) | 0.15 |
|  | Insurance status: other/unknown |  |  | -1.93 (-8.08, 4.23) | 0.54 |
|  | Race: White (ref) |  |  | 1 |  |
|  | Race: Black |  |  | 1.60 (-2.18, 5.38) | 0.41 |
|  | Race: other/unknown |  |  | 3.93 (-0.09, 7.96) | 0.06 |
|  | Sex: female (ref) |  |  | 1 |  |
|  | Sex: male |  |  | 1.10 (-1.36, 3.56) | 0.38 |
|  | Ever smoking status: no (ref) |  |  | 1 |  |
|  | Ever smoking status: yes |  |  | -0.38 (-2.73, 1.98) | 0.75 |
|  | Age |  |  | 0.04 (-0.04, 0.12) | 0.32 |
|  | BMI |  |  | 0.11 (-0.04, 0.26) | 0.15 |
|  | Hypertension: no (ref) |  |  | 1 |  |
|  | Hypertension: yes |  |  | 0.44 (-2.59, 3.46) | 0.78 |
|  | Diabetes: no (ref) |  |  | 1 |  |
|  | Diabetes: yes |  |  | -1.88 (-5.63, 1.86) | 0.32 |
|  | Coronary artery disease: no (ref) |  |  | 1 |  |
|  | Coronary artery disease: yes |  |  | -4.56 (-12.24, 3.13) | 0.24 |
|  | Depression/anxiety: no (ref) |  |  | 1 |  |
|  | Depression/anxiety: yes |  |  | 2.87 (0.42, 5.33) | 0.02 |
|  |  |  |  |  |  |
| **Subset with primary diagnosis = neurological (n=180)** |  |  |  |  |  |
|  | ADI national ranking value | 0.03 (-0.06, 0.12) | 0.48 | 0.07 (-0.04, 0.17) | 0.2 |
|  | Insurance status: private/managed care (ref) |  |  | 1 |  |
|  | Insurance status: Medicaid |  |  | -0.46 (-7.30, 6.38) | 0.89 |
|  | Insurance status: Medicare |  |  | 0.66 (-4.58, 5.90) | 0.8 |
|  | Insurance status: other/unknown |  |  | -13.09 (-24.54, -1.65) | 0.03 |
|  | Race: White (ref) |  |  | 1 |  |
|  | Race: Black |  |  | -0.50 (-7.28, 6.27) | 0.88 |
|  | Race: other/unknown |  |  | 2.84 (-5.72, 11.41) | 0.51 |
|  | Sex: female (ref) |  |  | 1 |  |
|  | Sex: male |  |  | -2.13 (-6.17, 1.91) | 0.3 |
|  | Ever smoking status: no (ref) |  |  | 1 |  |
|  | Ever smoking status: yes |  |  | 1.32 (-2.69, 5.34) | 0.52 |
|  | Age |  |  | -0.07 (-0.24, 0.09) | 0.38 |
|  | BMI |  |  | -0.10 (-0.40, 0.20) | 0.5 |
|  | Hypertension: no (ref) |  |  | 1 |  |
|  | Hypertension: yes |  |  | 1.69 (-2.70, 6.07) | 0.45 |
|  | Diabetes: no (ref) |  |  | 1 |  |
|  | Diabetes: yes |  |  | -3.00 (-8.77, 2.77) | 0.31 |
|  | Coronary artery disease: no (ref) |  |  | 1 |  |
|  | Coronary artery disease: yes |  |  | -2.53 (-10.47, 5.40) | 0.53 |
|  | Depression/anxiety: no (ref) |  |  | 1 |  |
|  | Depression/anxiety: yes |  |  | -0.65 (-5.28, 3.99) | 0.78 |
|  |  |  |  |  |  |
| **Subset with primary diagnosis = benign (n=345)** |  |  |  |  |  |
|  | ADI national ranking value | 0.03 (-0.02, 0.08) | 0.21 | 0.04 (-0.02, 0.10) | 0.15 |
|  | Insurance status: private/managed care (ref) |  |  | 1 |  |
|  | Insurance status: Medicaid |  |  | -2.14 (-6.41, 2.12) | 0.32 |
|  | Insurance status: Medicare |  |  | -2.53 (-6.27, 1.22) | 0.19 |
|  | Insurance status: other/unknown |  |  | -0.14 (-7.47, 7.18) | 0.97 |
|  | Race: White (ref) |  |  | 1 |  |
|  | Race: Black |  |  | -0.65 (-4.62, 3.31) | 0.75 |
|  | Race: other/unknown |  |  | -1.60 (-8.59, 5.38) | 0.65 |
|  | Sex: female (ref) |  |  | 1 |  |
|  | Sex: male |  |  | 0.98 (-1.63, 3.60) | 0.46 |
|  | Ever smoking status: no (ref) |  |  | 1 |  |
|  | Ever smoking status: yes |  |  | 1.19 (-1.36, 3.74) | 0.36 |
|  | Age |  |  | -0.06 (-0.17, 0.04) | 0.26 |
|  | BMI |  |  | -0.15 (-0.32, 0.03) | 0.11 |
|  | Hypertension: no (ref) |  |  | 1 |  |
|  | Hypertension: yes |  |  | 0.13 (-2.63, 2.89) | 0.93 |
|  | Diabetes: no (ref) |  |  | 1 |  |
|  | Diabetes: yes |  |  | 0.17 (-4.04, 4.37) | 0.94 |
|  | Coronary artery disease: no (ref) |  |  | 1 |  |
|  | Coronary artery disease: yes |  |  | 4.04 (-1.81, 9.88) | 0.18 |
|  | Depression/anxiety: no (ref) |  |  | 1 |  |
|  | Depression/anxiety: yes |  |  | 2.20 (-0.79, 5.20) | 0.15 |
|  |  |  |  |  |  |
| **Subset with primary diagnosis = oropharyngoesophageal or dysphagia (n=58)** |  |  |  |  |  |
|  | ADI national ranking value | 0.15 (0.03, 0.28) | 0.02 | 0.19 (-0.01, 0.38) | 0.06 |
|  | Insurance status: private/managed care (ref) |  |  | 1 |  |
|  | Insurance status: Medicaid |  |  | -2.00 (-14.72, 10.73) | 0.75 |
|  | Insurance status: Medicare |  |  | 3.13 (-8.11, 14.36) | 0.57 |
|  | Insurance status: other/unknown |  |  | -6.18 (-32.45, 20.10) | 0.63 |
|  | Race: White (ref) |  |  | 1 |  |
|  | Race: Black |  |  | -2.94 (-16.51, 10.64) | 0.66 |
|  | Race: other/unknown |  |  | -5.08 (-19.61, 9.45) | 0.48 |
|  | Sex: female (ref) |  |  | 1 |  |
|  | Sex: male |  |  | -2.73 (-11.72, 6.27) | 0.54 |
|  | Ever smoking status: no (ref) |  |  | 1 |  |
|  | Ever smoking status: yes |  |  | -3.82 (-12.08, 4.45) | 0.35 |
|  | Age |  |  | -0.07 (-0.34, 0.21) | 0.62 |
|  | BMI |  |  | -0.43 (-1.13, 0.27) | 0.22 |
|  | Hypertension: no (ref) |  |  | 1 |  |
|  | Hypertension: yes |  |  | -0.35 (-9.93, 9.23) | 0.94 |
|  | Diabetes: no (ref) |  |  | 1 |  |
|  | Diabetes: yes |  |  | 3.47 (-7.93, 14.87) | 0.54 |
|  | Coronary artery disease: no (ref) |  |  | 1 |  |
|  | Coronary artery disease: yes |  |  | 5.31 (-11.97, 22.59) | 0.53 |
|  | Depression/anxiety: no (ref) |  |  | 1 |  |
|  | Depression/anxiety: yes |  |  | 5.72 (-3.27, 14.70) | 0.2 |
|  |  |  |  |  |  |
| **Subset with primary diagnosis = dyspnea/cough/throat irritation (n=143)** |  |  |  |  |  |
|  | ADI national ranking value | 0.05 (-0.008, 0.11) | 0.09 | -0.01 (-0.07, 0.06) | 0.77 |
|  | Insurance status: private/managed care (ref) |  |  | 1 |  |
|  | Insurance status: Medicaid |  |  | 12.12 (6.62, 17.62) | <0.001 |
|  | Insurance status: Medicare |  |  | 1.38 (-2.97, 5.74) | 0.53 |
|  | Insurance status: other/unknown |  |  | 0.16 (-16.00, 16.31) | 0.98 |
|  | Race: White (ref) |  |  | 1 |  |
|  | Race: Black |  |  | 0.19 (-5.25, 5.62) | 0.95 |
|  | Race: other/unknown |  |  | 2.84 (-2.42, 8.10) | 0.29 |
|  | Sex: female (ref) |  |  | 1 |  |
|  | Sex: male |  |  | -1.38 (-4.79, 2.03) | 0.42 |
|  | Ever smoking status: no (ref) |  |  | 1 |  |
|  | Ever smoking status: yes |  |  | 2.62 (-0.68, 5.92) | 0.12 |
|  | Age |  |  | 0.14 (-0.01, 0.28) | 0.07 |
|  | BMI |  |  | 0.09 (-0.17, 0.35) | 0.49 |
|  | Hypertension: no (ref) |  |  | 1 |  |
|  | Hypertension: yes |  |  | -3.38 (-7.28, 0.53) | 0.09 |
|  | Diabetes: no (ref) |  |  | 1 |  |
|  | Diabetes: yes |  |  | -2.29 (-7.74, 3.16) | 0.41 |
|  | Coronary artery disease: no (ref) |  |  | 1 |  |
|  | Coronary artery disease: yes |  |  | -2.93 (-12.70, 6.85) | 0.55 |
|  | Depression/anxiety: no (ref) |  |  | 1 |  |
|  | Depression/anxiety: yes |  |  | 4.24 (0.16, 8.32) | 0.04 |

* A Bonferroni adjusted p-value of 0.017 was considered statistically significant

** One patient with unknown sex was excluded from these models

*** Includes primary diagnosis categories: cancer, infectious/inflammatory, and bilateral vocal fold paralysis

ADI: Area Deprivation Index; BMI: Body Mass Index; RSI: Reflux Severity Index; VHI-10: Voice Handicap Index-10; LPRD: Laryngopharyngeal Reflux Disease; GERD: Gastroesophageal Reflux Disease
